# The Expanding Horizon of Neural Stimulation for Hyperkinetic Movement Disorders

**DOI:** 10.3389/fneur.2021.669690

**Published:** 2021-05-14

**Authors:** Anna Latorre, Lorenzo Rocchi, Anna Sadnicka

**Affiliations:** ^1^Department of Clinical and Movement Neurosciences, University College London, London, United Kingdom; ^2^Department of Medical Sciences and Public Health, University of Cagliari, Cagliari, Italy; ^3^Motor Control and Neuromodulation Group, St George's University of London, London, United Kingdom

**Keywords:** Parkinson's disease, tremor, dystonia, Gilles de la Tourette syndrome, deep brain stimulation, non-invasive brain stimulation, peripheral stimulation, machine learning

## Abstract

Novel methods of neural stimulation are transforming the management of hyperkinetic movement disorders. In this review the diversity of approach available is showcased. We first describe the most commonly used features that can be extracted from oscillatory activity of the central nervous system, and how these can be combined with an expanding range of non-invasive and invasive brain stimulation techniques. We then shift our focus to the periphery using tremor and Tourette's syndrome to illustrate the utility of peripheral biomarkers and interventions. Finally, we discuss current innovations which are changing the landscape of stimulation strategy by integrating technological advances and the use of machine learning to drive optimization.

## Introduction

The application of electricity to influence physiological function of the nervous system dates back to the late eighteenth century (1), but it has been only in the last decades that, thanks to technological advancement, a substantial interest in methods for non-invasive and invasive neural stimulation has developed. The common denominator of these applications is to interact with ongoing neural activity to produce measurable effects on behavior. In this article we showcase the miscellany of stimulation techniques available and discuss some of the methods employed to extract features from central and peripheral recordings. We detail how advances in technology are likely to transform the management of hyperkinetic movement disorders in the future.

## Central Biomarkers And Stimulation

Central stimulation techniques include several non-invasive methods such as transcranial magnetic stimulation (TMS) (2) and transcranial current stimulation with either direct (TDCS) or alternating current (tTACS) (3, 4). To date, non-invasive neurostimulation techniques have been employed in hyperkinetic movements disorders with variable outcomes (5, 6). For example, there have been some promising results for dystonia, chorea, dyskinesia in Parkinson's disease and tics. However, overall, according to current evidence-based guidelines, there is not enough evidence to use non-invasive stimulation as a routine treatment or add-on therapy for hyperkinetic movement disorders (7). The reasons for this include insufficient data availability due to the heterogeneity of the protocols applied, the lack of a mechanistic understanding/rationale for choosing the cortical target and technical issues (5, 8).

An alternative approach is deep brain stimulation (DBS), a neurosurgical procedure that allows targeted circuit-based neuromodulation (9). Although invasive, its benefit and risk profiles are well-established and its efficacy compared to non-invasive brain stimulation in treating hyperkinetic movement disorders is beyond question. DBS is commonly used in the treatment of PD, tremor and dystonia (9, 10). DBS may also be effective in tardive dyskinesia, chorea (including Huntington's disease and neuroacanthocytosis), myoclonus–dystonia syndrome, Tourette's syndrome (TS) and other tremor syndromes (such as orthostatic tremor and Holmes' tremor) (11).

Despite the widespread application of non-invasive and invasive techniques and the successful application of DBS for the treatment of several conditions, knowledge about their interaction with ongoing brain activity, which ultimately cause behavioral effects, is scarce. This is considered an important limitation, since understanding how to tune brain stimulation in order to efficiently interact with neuronal processes could enhance its efficacy (12). For example, it has been speculated that at least part of the variability in the effects elicited by repetitive electric/magnetic pulses is due to the fact that these activate neurons in different functional states (12, 13). Therefore, it is reasonable to hypothesize that forms of brain stimulation impinging on pre-selected brain states, and thus of neuronal excitability, would lead to more consistent and predictable effects (14). This strategy entails the use of a readout, which can be analyzed online and guide the stimulation based on specific features, a principle known as closed-loop stimulation.

A frequently used readout is electrical brain activity, measured in the form of oscillations in different frequency bands. Oscillatory electrical activity occurs in the brain when groups of neurons synchronize their firing, and plays a crucial role in regulating brain function, in physiological and pathological contexts. When sufficiently large populations of interconnected neurons are synchronized, brain oscillations are observable as local field potentials (LFPs) and with surface electroencephalography (EEG) and magnetoencephalography (MEG) recordings, thereby reflecting instantaneous markers of neuronal networks excitability (15, 16).

Both power and phase have been used to guide rTMS and DBS, in the experimental and clinical settings. Several TMS studies have used EEG-triggered TMS, applied on the primary motor cortex (M1), based on the phase of the central mu rhythm. In general, its negative phase has been reported to correspond to a state of increased excitability of the primary motor cortex (M1), reflected by increased amplitude of motor evoked potentials (MEP) (13, 17) and transcranial evoked potentials (18). Targeting differential brain states by means of phase estimation has also been associated to increased effectiveness in inducing synaptic plasticity: Baur and colleagues suggested that 1 Hz rTMS induces stronger long-term depression-like plasticity in M1 if pulses are delivered at the positive peak of the mu rhythm, when compared to 1 Hz rTMS given at a random phase. By contrast, when stimuli correspond to the negative peak of mu rhythm, a trend toward long-term potentiation-like plasticity occurs (19). Whether this greater effects in modulation of brain activity can translate into more effective therapies is yet to be established. A promising result in this regards comes from the work by Zrenner and colleagues, which suggests that triggering TMS at the negative peak of instantaneous alpha oscillations in the dorsolateral prefrontal cortex in patients with resistant major depressive disorder reduces resting-state alpha activity, with an effect size larger than rTMS given at random alpha phase or than a variant of intermittent theta-burst stimulation (20), thus showing potential for effective neuromodulation in these patients.

Closed-loop application of DBS (often called adaptive DBS, aDBS) in PD usually involves modulation of the stimulation pattern based on real-time estimation of LFPs power in the beta frequency band, as beta activity has been shown to correlate with bradykinesia and rigidity (21–24). Several studies comparing adaptive and conventional DBS in PD have suggested that the former might present some clinical and technical advantages. aDBS has been found to have greater effectiveness in reversing motor deficits, although small methodological differences might explain these results (25). A technical advantage of aDBS is represented by battery saving, which is greater than conventional DBS, particularly in the on drug state (26, 27), even allowing for the extra signal processing necessary for aDBS (28). Some evidence also points toward a more favorable pattern of side effects with aDBS, represented by a lower incidence of dysarthria (26, 29). aDBS based on phase of the recorded signals has received less attention so far. Rosin and coworkers (30) demonstrated, in a non-human primate model of PD, that phase-based aDBS was more effective at attenuating motor symptoms compared to conventional DBS when applying brief, high frequency bursts of simulation to the globus pallidus pars interna 80 ms after the detection of spikes in single neurons recorded in the ipsilateral M1. The time delay was critical in improving motor impairment and corresponded to the cycle of the 9–15 Hz beta band oscillations typical of this model. Other studies showed that brief bursts of stimulation pulses asynchronously delivered to the STN lead to improvement of motor symptoms in primates and humans (31, 32), possibly as a consequence of neuronal phase resetting followed by plastic changes in local neural circuits.

Adapting brain stimulation to a physiological readout can be challenging for a number of reasons. For instance, the oscillation of interest needs to be ample enough to ensure a good signal to noise ratio (33, 34), and to be as closely related as possible to the physiological or pathological phenomenon under investigation. As outlined in the next section, tremor conditions offer pathophysiological markers which mostly satisfy these requirements and can thus be considered good candidates for closed-loop stimulation applications.

## Peripheral Biomarkers And Stimulation

A neurological disorder which particularly lends itself to adaptive stimulations techniques is central tremor. Regardless of the underlying disease, it entails a rhythmic muscle contraction caused by synchronous discharges generated in the central nervous system (35, 36). As such, the neural oscillatory activity responsible for the rhythmic, involuntary movement, could be reliably estimated by using muscle activity or joint acceleration as readouts. Thus, the peripheral oscillation (1) can be used as a control signal to regulate the stimulation of the neural population, (2) can be easily measured to monitor the effect of brain stimulation, (3) can inform on rhythmic activity of neurons in specific brain areas. Central tremors, such as ET and tremor in PD, are thought to be driven by periodic oscillatory activity generated by an unstable loop circuit within the central nervous system or by a nucleus with spontaneous rhythmic activity, arising from ion channels dynamics through inhibition-induced excitation (37). For instance, in ET, firing patterns of neurons in the thalamic ventralis intermedius nucleus (VIM), which receives substantial input from the cerebellum, are coherent with peripheral tremor (38). The thalamus is one of the main nodes of the cerebello-thalamo-cortical (CTC) network, i.e., an anatomical-functional source with a crucial role in ET generation (39). Although circuitry underpinning parkinsonian rest tremor and ET differs in its functional characteristics and connections (40), the CTC network is also implicated in the pathophysiology of tremor in PD, together with abnormal oscillatory activity within the basal ganglia (41). It is thought that phase alignment between the different neural populations of the network determines the degree of synchrony and therefore the efficacy of the generated pattern (42). Conversely, when synchronization is disturbed, effective connectivity would decrease, because synaptic input is more likely to arrive at random phases (42). This implicates that the interaction between neural populations could be modified dynamically by disrupting the phase alignment between different brain regions; this in turn, could potentially weaken the neural communication and its outcome, i.e., tremor. It is for this reason that high frequency thalamic DBS is used for the treatment of tremor; in fact, compared to lower-frequency DBS, it has an increased probability of stimulating the underlying pathological oscillation at the right time, thus disrupting the relay of this oscillation to motor cortex and control tremor (43).

Based on this principle, it has been explored whether stimulating at a selected tremor phase would lead to a greater oscillation disruption and consequent tremor control. With this intent, DBS stimulation has been delivered at specific phases of the tremor cycle in PD patients with subthalamic or ventrolateral thalamic DBS and ET patients with ventrolateral thalamic DBS (40, 44). Patients were stimulated with a frequency at the nearest integer frequency of their tremor, but stimulation was not actively locked to the tremor phase; therefore, stimulation and tremor were allowed to drift in and out of phase, revealing instantaneous effects of stimulation timing. While in both PD and ET DBS significantly entrained tremor, tremor amplitude was modulated only in ET, depending on the timing of stimulation pulses with respect to the tremor cycle. Interestingly, prominent tremor suppression was observed when stimulation was delivered at phases promoting suppression over several tremor cycles (44), indicating a cumulative effect which was likely induced by mechanisms of short-term (spike-timing dependent) plasticity (44). The lack of amplitude modulation in PD might be related to its broad frequency-amplitude tolerance (tremor amplitude remains unchanged despite spontaneous changes in instantaneous tremor frequency), but also potentially to stimulation location (see below) or tremor circuit complexity (40). This approach has been refined by tailoring the stimulation timing to a specific tremor phase in a closed-loop fashion. In this case, in each tremor cycle, a burst of high frequency DBS pulses (as typically used to control tremor) is delivered to the ventrolateral thalamus, phase-locked to the tremor phase during which amplitude suppression was most effective. This induced a clinically significant tremor relief (up to 87% tremor suppression compared to baseline, in the absence of DBS stimulation) in selected ET patients, with the benefit of delivering less than half the energy of conventional high frequency stimulation (45).

TACS is another well-established, non-invasive brain stimulation technique able to entrain tremor by interacting with its pathological neural network. In the pioneering study of Brittain et al., tACS was applied over the primary motor cortex of PD patients, at tremor frequency and phase-locked to the on-going tremor (recorded by an accelerometer), inducing almost 50% average reduction in rest tremor amplitude if delivered during the optimal phase for tremor suppression (46). Differently from DBS (40), in PD tACS had a positive effect on tremor amplitude, maybe because of the different location where stimulation was applied (primary motor cortex instead of basal ganglia). The use of tACS to control tremor in a recent study in ET has also provided potential mechanisms underlying tremor suppression (47). In this study, Schreglmann and colleagues investigated whether cerebellar tACS, phase-locked to tremor oscillation, is able to perturbate synchronous cerebellar activity and disrupt the CTC network oscillations to control ET. The main novelty of the study is that, to enable phase-locking of stimulation to oscillatory activity, the authors developed a strategy to compute in real-time the instantaneous phase of oscillatory signals by the endpoint-corrected Hilbert transform, to overcome the characteristic Gibbs distortion that has made it impossible before to precisely compute instantaneous tremor phase and amplitude. Peripheral tremor was used as a proxy for central oscillatory activity, providing a non-invasive means of identifying phase dependency for cerebellar phase cancellation. Eight different patterns of stimulating current were delivered, six sinusoidal at phase lags (0°, 60°, 120°, 180°, 240°, 300°), a control sinusoidal at the tremor frequency without phase locking, and a sham. The results demonstrated that phase-locking cerebellar stimulation can efficiently suppress ET amplitude within a few seconds. The phases that were effective in suppressing the tremor varied among participants. Interestingly, as noted in previous studies, tremor amplitude continued to drop after the end of the stimulation period. Further analysis also showed that change in tremor amplitude was associated with a change in temporal coherence, suggesting that stimulation that disrupts the temporal coherence can reduce tremor severity. Using a neurophysiological model of the CTC network under ET condition, the authors suggested that tremor suppression might be related to a timely perturbation of the generation of aberrant complex spikes in the Purkinje cells, therefore disrupting the synchronous activity that generate oscillations in the olivocerebellar loop (47, 48).

The possibility of attenuating tremor by peripheral stimulation has also been explored (49, 50), based on the rationale that peripheral stimulation can induce central activity in brain regions, including the VIM (51). Preliminary results, on a limited number of subjects, have shown the ability of this technique to modulate tremor with both open- and closed-loop stimulation, stimulating median and ulnar nerves at the wrists (49, 50).

In contrast with the purer motor syndrome of tremor, TS is a neurodevelopment disorder characterized by the occurrence of vocal and motor tics of childhood onset and associated with neuropsychiatric features such as obsessive compulsive disorder and attention deficit hyperactivity disorder. In ~90% of individuals tics are preceded by premonitory sensory and urge phenomena (52). Expressing tics gives temporary relief from such urge sensations and many patients believe they would not exhibit tics if they did not experience urges (53). Cortical oscillations in the mu and beta frequency bands may be promising neural markers of Tourette's Syndrome. Mu rhythm are synchronized patterns of electrical activity in cortical areas directly involved in voluntary movement, at a frequency similar to the alpha rhythm which occurs in the resting visual cortex. In health, mu oscillatory power is suppressed when a person performs a motor action and is most prominent when the body is physically at rest. Both mu and beta are thought to be relevant to the occurrence of tics with cortical oscillatory signals over the supplementary motor area abnormal ahead of tic execution (53, 54). It has previously been shown that rTMS causes local entrainment of cortical oscillations (55). Jackson and coworkers therefore investigated whether median nerve stimulation could be used to entrain brain oscillations linked to suppression of movement and influence tic expression. Firstly, they were able to show that rhythmical 12 Hz electrical stimulation to the right wrist produced a sustained increase in 12 Hz power and phase synchrony in EEG recorded from contralateral sensorimotor areas (53). Then in 16 patients exposed to pulses of rhythmical mu band stimulation they were able to show that both tic frequency and tic intensity were significantly reduced when compared to epochs of no stimulation. Effects were quantified by blind analysis of video recordings and subjective reporting, with a close correspondence between the two (53). The clinical effect also appeared to be mechanistically independent to attentional focus as performance on simultaneous cognitive tasks did not change.

## Future Directions

### Novel Classification

The approaches outlined in previous sections can be used to exemplify different strategies available to treat the hyperkinetic movement disorders. One strategy attempts to interact with causative neural biomarkers in approaches that will be specific to a particular disease. Thus, in PD, relative to many other movement disorders we are have a good approximation of the chain of disease at multiple levels of investigation. Increased neural synchrony in the beta frequency is a likely downstream repercussion of the primary neurodegenerative change; yet, by using this as an input for adaptive stimulation, we believe we are interacting with a causal or essential neural mechanism. With such a disease-specific intervention, further understanding of pathophysiology will allow the development of useful biomarkers to dictate stimulation. This is conceptually distinct to a syndromic approach in which similarities in the final pathway of a movement disorder and/or shared kinematic features allow strategies to be developed that have utility across a range of etiologies. To date, such an approach is most readily exemplified in strategies of tremor treatment. There is also the overlap of our archetypical movement disorders: for example, dystonia is often associated with tremor, and choreiform-like movements complicate PD in the form of dyskinesias. It is therefore likely that we develop overlapping indications for many stimulation strategies that do not conform to descriptive neurological classifications.

**Figure 1:**
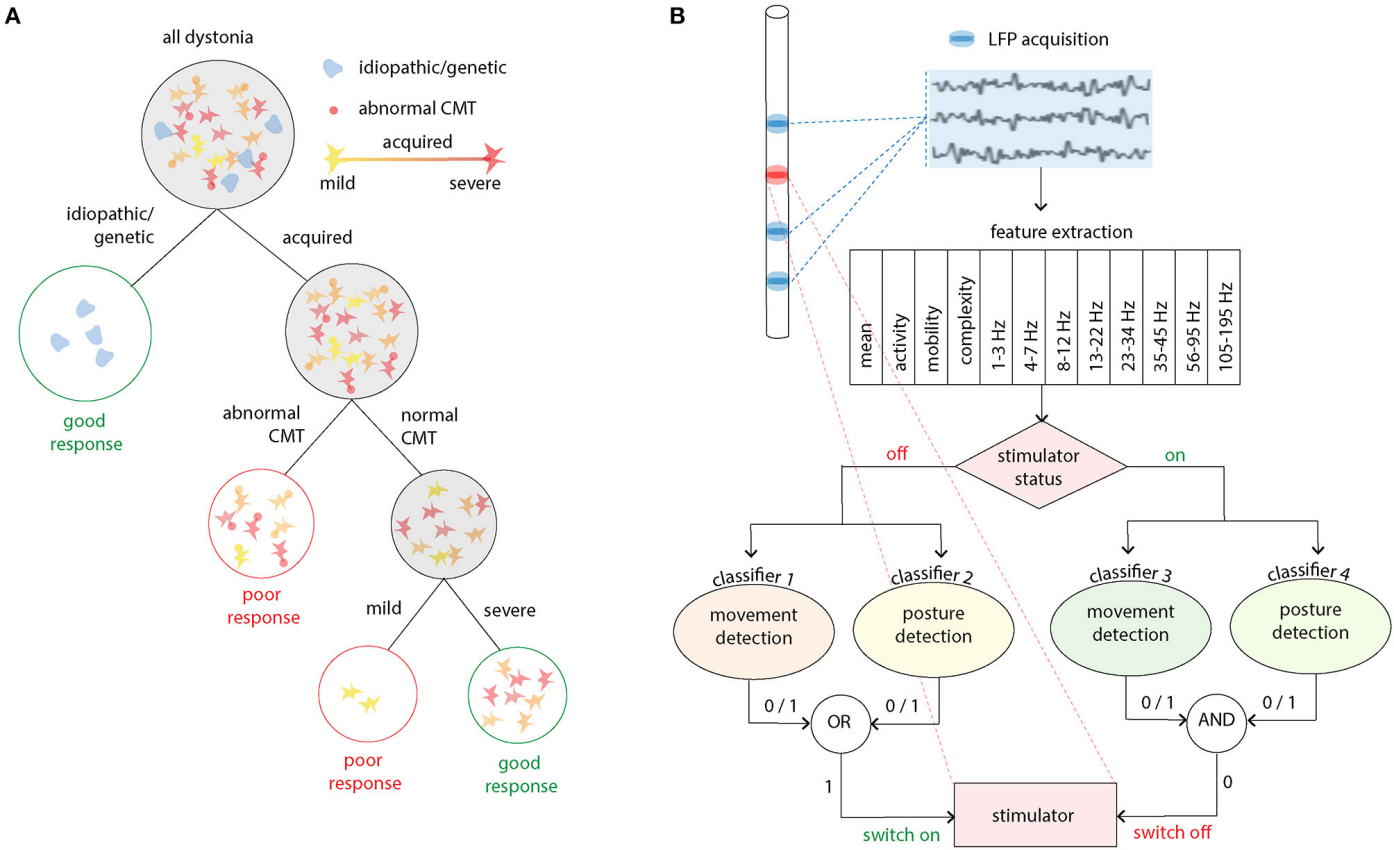
Applications for machine learning in deep brain stimulation. **(A)** A decision tree method was used to identify clinical demographics and neurophysiological markers which predict good outcome in acquired childhood dystonia. In this study three nodes or levels of decision making were identified. Firstly, idiopathic and genetic dystonias should be recommended for deep brain stimulation as they are known to have a good response (>20% improvement in clinical scores). The middle node then examines whether the corticospinal tract is intact using abnormalities in the central motor conduction time (CMT) as the delineator. Finally, more severe disease is predictive of a good response [adapted from (56)]. **(B)** This panel exemplifies a real time closed loop algorithm which has been successfully used in essential tremor. Twelve features of the LFP are used to train four classifiers for two stimulator states (off/on) and two movement states (movement/posture detection). The presence (1) or absence (0) or the movement states dictates whether the stimulator state is changed [adapted from (57)].

The generation of response-led classifications is also an emergent theme and here the use of machine learning can guide development. Such an approach was recently used with good effect in childhood dystonia (56). In this study, six patient parameters (sex, etiology, baseline severity, cranial MRI and central motor conduction time and/or sensory evoked potential) were evaluated for their ability predict deep brain stimulation outcome using a decision tree supervised learning method (Figure 1A). This method prioritized clinical interpretability and evaluated all possible combinations of the six parameters (2^6^–1) for their ability to predict favorable clinical outcome. In the acquired group, both integrity of motor pathways (or sensory pathways in a subsequent analysis) and the severity of dystonia were important. Furthermore, since the full variation of performance for different dystonia severity cut-offs were known, such information could also feed into decision making in a patient specific manner. For example, if any clinical improvement was likely to have a large impact, no matter how small, then a lower sensitivity and specificity of the severity criterion could be selected. Conversely, if the patient, family and clinician wanted to take a more risk-adverse strategy with greater certainty of predicated outcome, then high sensitivity and specificity could be achieved by using a higher severity score as the cut-off for decision making. This study therefore started to dissect the determinants of variability in outcome in a diverse patient group with a very modest palette of input data.

### Novel Biomarkers

Most biomarkers to date that have been used for adaptive stimulation in movement disorders are those directly related to neurophysiological signals such as LFPs and electromyograms or inertial sensors such as accelerometers. Most analysis has concentrated on the use of specific signal features, such as power or phase in the beta frequency band. However, in terms of information content, the input data are usually incredibly rich, and such unidimensional metrics capture only a fraction of their potential. In the future, machine learning methods are likely to be widely used to optimize feature extraction for neural stimulation.

Such an approach has recently been used in patients with ET with encouraging results. Firstly, Tan et al. investigated whether voluntary movement and the presence of postural tremor could be decoded from LFPs recorded simultaneously from electrodes implanted in the motor thalamus for stimulation. A logistic regression model was able to decode both voluntary movements and the presence of postural tremor with good sensitivity and specificity and, although beta frequency bands (13–23 Hz) and theta frequency bands (4–7 Hz) contributed most to the decoding, the incorporation of different frequency bands using a machine learning approach increased the accuracy of decoding (58).

In a follow-up work, the group therefore studied the use of thalamic LFPs for real-time closed-loop DBS in ET (Figure 1B). DBS electrodes were inserted into the VIM and zona incerta (ZI), and stimulation and LFPs recordings occurred simultaneously (57). Twelve features in time and frequency domains were extracted from the bipolar LFPs recorded from VIM-ZI thalamus. As stimulation induced changes in the neural activities and artifacts in the recording, model parameters were trained for different simulation states (on or off) and trained to detect patient specific classifiers for voluntary movements and posture decoding (a total of four classifiers). As the stimulation was controlled automatically by the system, the status of the stimulator at any moment was also recorded by the program. The performance of several classification methods was tested and linear methods outperformed other methods that take into account non-linear relationships (advantageous as linear models tend to require less data to train models and have a less demanding processing requirement for online decoding). Compared with continuous stimulation, a similar amount of tremor suppression was achieved whilst delivering <40% of the energy required for continuous stimulation (57). Such approaches are enormously exciting as devices with the capacity for chronic sensing and bidirectional communication become available.

### Novel Stimulation Capabilities

Novel hardware is currently flooding into clinical practice (10). For example, in the field of DBS, spatial selectivity is enhanced through higher resolution electrodes and the increasing range of stimulation options (unipolar, bipolar, interleaving stimulation, multiple-level stimulation, directional current flow) (10). Dynamically there is likely to be a move away from monotonic high-frequency stimulation toward temporal patterning informed by dynamics in neural circuits and symptoms. The continued focus on miniaturization will drive innovations in device design; cardiac pacemakers can now be implanted through endovascular techniques and such routes are also being explored for neuromodulation, which would avoid the need for cranial burr holes and tissue-disrupting lead insertion (59). Minimally invasive methods are in development, such as transcranial ultrasound, which enable a “non-invasive” ablation of neural circuits, such as those in the thalamus for tremor (48). It may also be possible to use such modalities in real time to modulate cortical and subcortical circuits providing many of the benefits of DBS without the requirement of cranial surgery (60). Peripherally, there is also an explosion of non-invasive, wearable, and compact devices both for monitoring symptoms and potential therapeutic intervention. The use of secure telemetry allows continuous wireless upload of data allowing more complex control on multiple timescales. By using off-the-body local and distributed cloud computing systems, such data can be fed into machine-learning methods to provide summaries that aid decision-making.

### Connected Open Source Community

We are therefore likely to move away from empirical clinician designed stimulation to high-fidelity models of the relationship between the pattern of stimulation and changes in disease/symptoms state. Optimal development will hinge on a close partnership between the patient community, their clinicians, scientists and industry. Any telemetry method also requires great care in security and risk management to ensure patient safety and minimize the threat of malicious hacks. A commitment of the community to open science will also democratize and increase the speed of advances with high uptake of currently available initiatives such as LEAD-DBS (61). This freely available toolbox can be downloaded to allow electrode reconstructions and computer simulations based on post-operative MRI and CT imaging.

## Conclusions

We have overviewed the use of neurostimulation techniques in hyperkinetic movement disorders and focused on conditions in which physiological biomarkers are available to guide stimulation protocols. We believe that advances in technology and methods will transform the management of hyperkinetic movement disorders over the next few decades. We are optimistic that the future challenge of providing individualized stimulation highly responsive to clinical state for a full range of hyperkinetic movement disorders with minimal risk is achievable.

## Author Contributions

All authors contributed to the article and approved the submitted version.

## Conflict of Interest

The authors declare that the research was conducted in the absence of any commercial or financial relationships that could be construed as a potential conflict of interest.
